# Faecal carriage of multidrug-resistant Enterobacterales in hospitalisaed and outpatient children in northern India: A cross-sectional study

**DOI:** 10.4102/ajlm.v15i1.3027

**Published:** 2026-07-31

**Authors:** Akansha Goyal, Jannatbir Kaur, Sapna Chauhan

**Affiliations:** 1 Department of Microbiology, Muzaffarnagar Medical College, Muzaffarnagar, India

**Keywords:** asymptomatic infections, intestinal microbiome, multidrug-resistant organisms, drug resistance, multiple, Enterobacterales

## Abstract

**Background:**

Asymptomatic intestinal carriage of multidrug-resistant (MDR) Enterobacterales in children is increasingly recognised as an important reservoir for transmission and subsequent infection. However, data from northern India remain limited, warranting evaluation of its burden and associated risk factors.

**Objective:**

To determine the prevalence of MDR Enterobacterales carriage among children and to identify associated risk factors, including recent hospitalisation, antimicrobial exposure, and hygiene practices.

**Methods:**

This cross-sectional study was conducted at a tertiary care hospital in northern India from January to June 2025. Children aged ≤ 14 years were enrolled after obtaining informed consent from parents or legal guardians. A total of 340 stool samples were processed using standard microbiological methods. Bacterial identification was performed using conventional biochemical tests, and antimicrobial susceptibility testing was carried out according to Clinical and Laboratory Standards Institute guidelines, 2025 edition. Multidrug-resistant was defined as resistance to at least one agent in three or more antimicrobial classes. Demographic and clinical data were analysed using SPSS version 25.0; *p* < 0.05 was considered statistically significant.

**Results:**

Among 340 enrolled children, Enterobacterales were isolated from 309 (90.9%). Multidrug-resistant Enterobacterales were identified in 87 children, yielding a prevalence of 28.2% among Enterobacterales-positive children and 25.6% overall. *Escherichia coli* (66.7%) and *Klebsiella* spp. (33.3%) were the predominant MDR organisms. Extended-spectrum β-lactamase production was detected in 18.1% of isolates. Multidrug-resistant carriage was significantly higher among inpatients (*p* < 0.001).

**Conclusion:**

Asymptomatic carriage of MDR Enterobacterales is common, particularly among hospitalised children, highlighting the importance of strengthened antimicrobial stewardship and infection prevention strategies in paediatric care.

**What this study adds:**

This study demonstrates a high prevalence of asymptomatic multidrug-resistant Enterobacterales carriage among children in northern India, particularly among hospitalised children, highlighting the need for ongoing surveillance, infection prevention and antimicrobial stewardship.

## Introduction

The growing threat of antimicrobial resistance (AMR) is no longer restricted to pathogenic organisms alone; it has also become evident among the normal bacterial flora that inhabit the human body.^1^ Among these, intestinal commensals – particularly Enterobacterales – serve as important reservoirs of resistance genes. Although these bacteria may not cause immediate disease, their presence in the gut has significant clinical implications. Colonisation is now widely recognised as a crucial step preceding the development of infections, especially in vulnerable populations such as children and hospitalised patients.^2^

Importantly, asymptomatic intestinal carriage of antibiotic-resistant Enterobacterales can occur silently, often going undetected. This carriage may either precede or follow a clinical infection, and individuals can unknowingly become both recipients and transmitters of resistant strains.^3^ The presence of such organisms not only increases the risk of subsequent infections but also contributes to their persistence and spread within communities and healthcare settings.

When infections caused by these resistant organisms do occur, they are often difficult to treat. Antimicrobial resistant infections are associated with more severe illness, limited therapeutic options, treatment failure, and a significant increase in the duration of hospital stays. These factors contribute directly to a substantial rise in healthcare costs, placing additional burdens on both patients and health systems.^4^

The situation is further complicated by the global rise of multidrug-resistant (MDR) bacteria.

These organisms have spread rapidly across countries and continents, driven by mobile genetic elements and clonal dissemination. As a result, MDR infections have become a major clinical challenge, with rising rates of illness and death attributed to them. The consequences are felt not only in terms of patient outcomes but also in the economic impact of prolonged and intensive treatment regimens.^5^

While much attention has been directed toward treating antimicrobial-resistant infections, far less emphasis has been placed on identifying individuals who silently carry these organisms.

In children, who frequently interact with healthcare environments and often receive antibiotics early in life, the gastrointestinal tract can become a reservoir for MDR bacteria without any clinical symptoms. This asymptomatic colonisation holds significant public health implications, as it may contribute to the spread of resistance within both community and hospital settings.

Understanding the patterns of MDR carriage in children is particularly relevant in regions such as North India, where high antibiotic use and limited infection control measures may further amplify the problem. However, region-specific data remain scarce, particularly among paediatric populations. A clearer picture of local carriage rates, along with contributing demographic and clinical factors, is essential to inform early prevention strategies. Data on intestinal carriage of MDR Enterobacterales in Indian paediatric populations remain relatively limited, particularly from northern India.

This study aims to fill that gap by assessing the prevalence of asymptomatic intestinal carriage of MDR Enterobacterales among children aged 14 years or younger. The primary objective is to estimate the proportion of children carrying these resistant organisms. Secondary objectives include identifying associated risk factors – such as recent hospitaliation, antibiotic exposure, and hygiene practices – and characterising the resistance profiles of the isolated strains. The findings from this study are intended to contribute to early detection and control efforts, ultimately helping to limit the silent spread of resistance in both clinical and community settings.

## Methods

### Ethical considerations

The study protocol received approval from the Institutional Ethics Committee. The ethical clearance number is MMC/IEC/2021/237. Prior to participation, written informed consent was obtained from the parents or legal guardians of all enrolled children.

### Study design, setting, and duration

A cross-sectional study was conducted in the Department of Microbiology at a tertiary care hospital in northern India over a 6-month period from January 2025 to June 2025.

### Sample size calculation

The sample size was calculated using the single population proportion formula^6^ in Equation 1:

$$n=\left(Z^{2}\times p\times (1-p)\right)/d^{2}$$ [Eqn 1]

Where:

- *n* = required sample size
- *Z* = standard normal deviate for 95% confidence (1.96)
- *p* = estimated prevalence from previous study (0.33)^7^
- *d* = margin of error (0.05)

Based on these parameters, the minimum required sample size was approximately 340. Hence, 340 children were included in the present study.

### Eligibility criteria

#### Inclusion criteria

- Children aged 14 years or younger, either hospitalised or visiting outpatient services.
- Consent provided by a parent or legal guardian.

#### Exclusion criteria

- Receipt of systemic antibiotics within the preceding 72 h prior to sample collection.
- Critical condition preventing sample collection.
- Refusal to provide informed consent.

### Study procedure

Following consent, demographic details (age, sex), clinical history (recent hospitalisation within the past 3 months, underlying comorbidities, and recent antimicrobial use), and relevant risk factors including caregiver-reported hand hygiene practices were documented using a structured case reporting form. Recent antibiotic use was defined as receipt of any systemic antibiotic within the preceding 3 months. The structured case reporting form used for data collection is provided as supplementary material (Supplementary File 1).

For outpatient participants, stool samples were collected during the outpatient visit or brought from home on the same day of consultation in sterile containers. For hospitalised children, samples were collected within 48 h of admission wherever feasible to minimise the effect of prolonged hospital stay on colonisation status at the time of sampling. For infants, samples were obtained using sterile swabs from soiled diapers. All samples were clearly labelled and transported to the microbiology laboratory within 2 h of collection.

If delays occurred, specimens were stored at 2 °C – 8 °C and processed within 4 h.

In the laboratory, stool samples were inoculated aseptically onto MacConkey agar (HiMedia Laboratories, Mumbai, India) and 5% sheep blood agar (HiMedia Laboratories, Mumbai, India). Plates were incubated aerobically at 37 °C for 18–24 h. MacConkey agar facilitated the isolation and differentiation of lactose-fermenting and non-lactose-fermenting Gram-negative bacilli, while blood agar was used to assess haemolytic properties and support the growth of other enteric flora. All microbiological procedures were performed in accordance with standard methods described in Koneman’s *Color Atlas and Textbook of Diagnostic Microbiology*.^8^

After incubation, both lactose-fermenting and non-lactose-fermenting colonies with morphology suggestive of Enterobacterales were selected. These colonies were subjected to Gram staining, revealing Gram-negative bacilli, and were further identified using a panel of standard biochemical tests, including:

- Indole test: Performed using Kovac’s reagent (HiMedia Laboratories, Mumbai, India) for detection of tryptophanase activity.
- Methyl red test: Performed using methyl red indicator (HiMedia Laboratories, Mumbai, India) to assess stable acid end-product formation.
- Voges–Proskauer test: Performed using Barritt’s reagents A and B (HiMedia Laboratories, Mumbai, India) for detection of acetoin production.
- Citrate utilisation test: Performed using Simmons citrate agar (HiMedia Laboratories, Mumbai, India) to determine citrate utilisation as the sole carbon source.
- Urease test: Performed using urea agar medium (HiMedia Laboratories, Mumbai, India) to detect urea hydrolysis.
- Triple sugar iron (TSI) agar: Used to assess carbohydrate fermentation, gas production, and hydrogen sulfide production (HiMedia Laboratories, Mumbai, India).
- Motility test: Bacterial motility was assessed by the hanging drop method.

All confirmed isolates were subjected to antimicrobial susceptibility testing using the Kirby–Bauer disk diffusion method on Mueller-Hinton agar (HiMedia Laboratories, Mumbai, India). Inocula were adjusted to 0.5 McFarland turbidity standards. Antibiotic disks were applied, and plates were incubated at 37 °C for 16–18 h. The antibiotics tested included ampicillin, amoxicillin–clavulanate, cefotaxime, ceftazidime, ceftriaxone, ciprofloxacin, gentamicin, amikacin, piperacillin–tazobactam, and imipenem. Disk potencies were as per Clinical and Laboratory Standards Institute M100 (2025). Zone diameters were interpreted according to Clinical and Laboratory Standards Institute guidelines (2025 edition).^9^

Detection of extended-spectrum β-lactamase (ESBL) production was performed using the combination disk method in accordance with Clinical and Laboratory Standards Institute M100 (2025) guidelines.^9^ Isolates showing reduced susceptibility to third-generation cephalosporins were tested using cefotaxime (30 μg) and cefotaxime–clavulanate (30/10 μg) disks. An increase of ≥ 5 mm in zone diameter in the presence of clavulanate compared with cefotaxime alone was interpreted as ESBL production.

Carbapenem resistance was determined using disk diffusion in accordance with Clinical and Laboratory Standards Institute M100 (2025) guidelines.^9^ Isolates showing resistance or intermediate susceptibility to imipenem were to be classified as carbapenem-resistant Enterobacterales.

Quality control for susceptibility testing was ensured using *Escherichia coli* ATCC 25922 as a reference strain.

Multidrug-resistant was defined as resistance to at least one agent in three or more antimicrobial categories, in accordance with the international expert proposal by Magiorakos et al.^10^ and as applied by Pathak et al.^11^

The microbiology laboratory follows standard quality assurance protocols in accordance with institutional laboratory practices.

### Statistical analysis

Collected data were entered into a spreadsheet and analysed using SPSS version 25.0 (IBM Corp., Armonk, New York, United States). Descriptive statistics were used to summarise demographic and clinical characteristics. Associations between potential risk factors and MDR carriage were assessed using the chi-square test. A *p*-value of less than 0.05 was considered statistically significant.

## Results

### Study population characteristics

A total of 340 children aged ≤ 14 years were enrolled in the study. Enterobacterales were isolated from 309 stool samples (90.9%), while 31 (9.1%) were negative. To ensure independence of observations and avoid duplication bias, only one representative Enterobacterales isolate per child was included in the final analysis.

Among the Enterobacterales-positive children (*n* = 309), 117 (37.9%) were inpatients and 192 (62.1%) were outpatients at the time of sampling. The cohort comprised 170 (55.0%) boys and 139 (45.0%) girls.

Age-wise distribution showed 56 (18.1%) children aged < 1 year, 83 (26.9%) aged 1–5 years, and 170 (55.0%) aged > 5 years.

### Organism distribution

Among the 309 isolates, *Escherichia coli* was the predominant organism, identified in 192 (62.1%) cases, followed by *Klebsiella* spp. in 99 (32.0%) and other Enterobacterales species in 18 (5.8%).

#### Prevalence of multidrug-resistant Enterobacterales

Among the 309 Enterobacterales-positive children, 87 (28.2%) harboured MDR isolates, while 222 (71.8%) were non-MDR. Within the MDR group (*n* = 87), *E. coli* accounted for 58 (66.7%) isolates and *Klebsiella* spp. for 29 (33.3%). No MDR was observed among other Enterobacterales species (0/18).

#### Association with demographic and clinical factors

The association between demographic and clinical factors and multidrug-resistant Enterobacterales carriage is presented in Table 1. Multidrug-resistant carriage differed significantly by patient setting. Multidrug-resistant Enterobacterales were detected in 54 (46.2%) of 117 inpatients compared with 33 (17.2%) of 192 outpatients (*χ*^2^ = 30.16, *p* < 0.001).

**TABLE 1:** Association of demographic and clinical factors with multidrug-resistant carriage among Enterobacterales-positive children (*N* = 309).

Variable	Total		MDR		Non-MDR		χ^2^ value	*p*
	*N*	%	*n*	%	*n*	%		
**Age group (years)**	-	-	-	-	-	-	10.360	0.006*
< 1	56	18.1	10	17.9	46	82.1	-	-
1–5	83	26.9	34	41.0	49	59.0	-	-
> 5	170	55.0	43	25.3	127	74.7	-	-
**Sex**	-	-	-	-	-	-	0.001	0.972
Male	170	55.0	48	28.2	122	71.8	-	-
Female	139	45.0	39	28.1	100	71.9	-	-
**Patient setting**	-	-	-	-	-	-	30.160	< 0.001*
Inpatient	117	37.9	54	46.2	63	53.8	-	-
Outpatient	192	62.1	33	17.2	159	82.8	-	-

Note: Chi-square test applied. Percentages are calculated within each category.

MDR, multidrug-resistant.

* , *p* < 0.05 was considered statistically significant.

Multidrug-resistant carriage did not differ by sex: 48 (28.2%) of 170 boys and 39 (28.1%) of 139 girls had MDR isolates (*χ*^2^ = 0.001, *p* = 0.972).

Multidrug-resistant carriage differed significantly across age groups: 10 (17.9%) of 56 children aged < 1 year, 34 (41.0%) of 83 aged 1–5 years, and 43 (25.3%) of 170 aged > 5 years had MDR isolates (*χ*^2^ = 10.36, *p* = 0.006).

Among MDR-positive children (*n* = 87), recent antibiotic use within the preceding 3 months was reported in 60 (69.0%), while 27 (31.0%) reported no such exposure. Comorbid conditions were present in 40 (46.0%) children with MDR isolates; malnutrition was most common, reported in 21 (52.5%) of 40, followed by prematurity in 8 (20.0%) of 40, congenital heart disease in 6 (15.0%) of 40, and neurological disorders in 5 (12.5%) of 40.

#### Hygiene practices

Based on the caregiver report among MDR-positive children (*n* = 87), poor hand hygiene was noted in 50 (57.5%), while good hand hygiene was reported in 37 (42.5%).

#### Extended-spectrum β-lactamase-producing Enterobacterales

Extended-spectrum β-lactamase production was detected in 56 (18.1%) of 309 isolates. All ESBL-producing isolates (*n* = 56) fulfilled MDR criteria (100%). Among the ESBL isolates (*n* = 56), *E. coli* accounted for 42 (75.0%) and *Klebsiella* spp. for 14 (25.0%).

Extended-spectrum β-lactamase-producing isolates were more common among inpatients: 38 (32.5%) of 117 inpatients versus 18 (9.4%) of 192 outpatients. Within the ESBL group (*n* = 56), 38 (67.9%) were from inpatients and 18 (32.1%) were from outpatients.

Among the 87 MDR isolates, 31 (35.6%) were non-ESBL producers. No carbapenem-resistant Enterobacterales were detected.

## Discussion

When viewed in the context of existing literature, the findings of this study align with global and regional concerns regarding intestinal colonisation by MDR Enterobacterales in paediatric populations. In the present cohort, an MDR carriage rate of 28.2% among Enterobacterales-positive children (25.6% overall) was identified, including a notable MDR prevalence among outpatients (17.2%), reflecting the expanding community interface of AMR in children.

In a hospital-based study, Mittal et al.^1^ documented increased faecal carriage of carbapenemase-producing Enterobacterales among ICU patients over time, with prolonged hospital stay and exposure to invasive interventions identified as notable contributors. Similarly, Saxena et al.^2^ found colonisation with carbapenem-resistant Enterobacterales to be significantly associated with severe clinical conditions and the use of steroids or broad-spectrum antibiotics in paediatric inpatients. These observations support the present study’s finding that hospitalisation was strongly associated with MDR colonisation in children.

Extended-spectrum β-lactamase production was identified in 18.1% of isolates, with all ESBL producers fulfilling MDR criteria, highlighting the substantial contribution of ESBL mechanisms to the overall resistance burden.

Islam et al.,^3^ examining healthy children across multiple sites in the United States, reported markedly lower rates of third-generation cephalosporin-resistant (3GCR) Enterobacterales and ESBL-producing Enterobacterales. Their analysis suggested that international travel was one of the few factors linked with higher carriage, in contrast to the current study, where recent healthcare contact and antibiotic use were more relevant. Importantly, travel-associated ESBL acquisition has been particularly described in travellers to low- and middle-income countries, which may partially explain geographic variability in colonisation dynamics. This difference may reflect geographic variations in antimicrobial usage and transmission dynamics. Differences in carriage rates may also reflect variations in over-the-counter antimicrobial access, water and sanitation infrastructure, animal contact, and infection prevention practices across settings.

A study by Msanga et al.,^4^ involving both HIV-positive and HIV-negative Tanzanian children, found that prior antibiotic use and household exposure to recently hospitalised individuals were associated with ESBL colonisation. Their findings resonate with the current study, where prior antibiotic exposure was common among MDR-positive children in the present study, further emphasising the impact of recent antimicrobial pressure on colonisation.

Kaarme et al.,^5^ investigating ESBL carriage in healthy Swedish preschoolers, observed a substantial increase in prevalence over time, with evidence of within-preschool transmission. Although their study involved a community-based population, the high rates observed in certain institutions mirror localised outbreaks, which are relevant when considering the potential for horizontal spread of MDR organisms in shared environments.

In Uganda, Salega et al.^12^ reported an elevated rate of ESBL-producing Enterobacterales among neonates, influenced by hospital-related factors such as antibiotic exposure and length of stay. These observations are consistent with the present study’s associations, suggesting that even short-term hospitalisation may significantly alter the intestinal microbiota of children in favour of resistant strains.

Sandhu et al.,^7^ in a study involving under-five children in India, identified nutritional status, prior antimicrobial use, and socioeconomic indicators as important correlates of resistant colonisation. These findings are reflective of broader determinants of AMR in low- and middle-income countries, and their relevance to community-based paediatric populations parallels the non-hospitalised group in the current study.

In Zimbabwe, Magwenzi et al.^13^ found a high baseline prevalence of ESBL and aminoglycoside-resistant Enterobacterales in paediatric inpatients, with additional acquisition during hospitalisation. This highlights the dynamic nature of colonisation during hospital stays and is consistent with patterns observed in the current cohort.

Finally, Yap et al.^14^ described worrying rates of MDR colonisation in hospitalised preterm infants in Malaysia, particularly involving resistance to polymyxins. The identification of complex resistance patterns in these young, vulnerable patients raises concerns about antimicrobial exposure early in life and supports broader findings suggesting that hospitalisation and empirical antibiotic treatment contribute to colonisation with difficult-to-treat pathogens.

In contrast, no significant association was observed with sex in the present cohort, suggesting that healthcare-related exposures may play a more dominant role than baseline demographic factors.

### Clinical implication

The silent intestinal carriage of MDR organisms in children presents a significant concern for both individual patient care and broader public health. Even in the absence of symptoms, colonised individuals can serve as sources of resistant pathogens within homes, schools, and healthcare settings. For clinicians, this highlights the importance of considering colonisation status – particularly in children with frequent healthcare interactions – when making decisions around empirical treatment and infection control. Strengthening antimicrobial stewardship and reinforcing basic hygiene measures in paediatric care could help reduce the risk of resistant organism spread and the burden of associated infections. The high proportion of ESBL-producing isolates further reinforces the clinical relevance of these findings.

### Future perspective

There is a growing need to explore the trajectory of AMR beginning early in life. Future research should prioritise longitudinal designs that track colonisation over time and examine its impact on health outcomes. Understanding the interplay between antimicrobial exposure, environmental conditions, microbiome development, and socioeconomic factors may uncover preventive opportunities. Incorporation of genomic and molecular tools could also shed light on resistance mechanisms and potential community transmission networks. Ultimately, integrating these insights into paediatric health policy and public health initiatives could shape more effective and sustainable resistance mitigation strategies.

### Strength and limitations

This study adds meaningful evidence from a population often under-represented in AMR surveillance. It provides a real-world snapshot of MDR Enterobacterales colonisation in both outpatient and hospitalised paediatric settings. However, several limitations must be acknowledged. First, the cross-sectional design captures colonisation at a single time point and therefore limits inference regarding temporal acquisition or causality. Second, hygiene practices were based on caregiver self-report and may be subject to reporting bias; caregiver hygiene may differentially influence younger children who are more dependent on caregiver support. Third, primary isolation was performed using MacConkey agar, and overgrowth by *E. coli* may have reduced detection of less predominant Enterobacterales species; the use of chromogenic or more selective media might have improved species recovery. Selective media for *Salmonella* spp. and *Shigella* spp. were not used, which may have further reduced recovery of these organisms. The absence of molecular characterisation restricts the depth of resistance profiling, and potential confounders such as environmental hygiene and caregiver practices were not fully explored. In addition, differences in baseline healthcare exposure between inpatient and outpatient groups may have influenced comparisons between these groups. Despite these limitations, the study offers valuable direction for more targeted and methodologically robust future investigations.

### Conclusion

Understanding asymptomatic colonisation by resistant bacteria in children is essential for developing a proactive response to AMR. As resistance continues to emerge even beyond hospital walls, early identification of risk factors and community-level interventions become increasingly important. This study reinforces the call for integrated surveillance, education, and stewardship efforts aimed at preserving antimicrobial efficacy for future generations.
